# Effect of Aspirin on Cell Growth of Human MG-63 Osteosarcoma Line

**DOI:** 10.1100/2012/834246

**Published:** 2012-05-02

**Authors:** E. De Luna-Bertos, J. Ramos-Torrecillas, O. García-Martínez, L. Díaz-Rodríguez, C. Ruiz

**Affiliations:** ^1^Biomedical Group (BIO277), Department of Nursing, Faculty of Health Sciences, University of Granada, 18071 Granada, Spain; ^2^Institute of Neuroscience, University of Granada, 18071 Granada, Spain

## Abstract

Nonsteroidal anti-inflammatory drugs (NSAIDs) are commonly used in bone tissue repair treatment for their pharmacological action. The objective of this study was to determine the effect of aspirin, on osteoblast growth, using MG63 cell line as osteoblast model. MTT spectrophotometry results showed that 20, 100, and 1000 *μ*M aspirin doses have an inhibitory effect on growth. Cell cycle analysis revealed that aspirin doses of 100 and 1000 *μ*M arrest the cell cycle in phase GO/G1. Parallel apoptosis/necrosis studies showed no changes in comparison to control cells after treatment with 1 or 10 *μ*M aspirin but a significantly increased percentage of cells in apoptosis at doses of 20, 100, and 1000 *μ*M. We highlight that treatment of osteoblast-like cells with 1000 *μ*M aspirin increased not only the percentage of cells in apoptosis but also the percentage of necrotic cells, which was not observed in aspirin treatments at lower doses.

## 1. Introduction

Nonsteroidal anti-inflammatory drugs (NSAIDs) are widely used in orthopaedic surgery and traumatology to reduce pain and inflammation. Animal and cell culture studies have shown that some NSAIDs have an adverse effect on bone tissue through regeneration delay [1–6].

The mechanism by which NSAIDs exert their action on cell growth is under debate. Some authors cite the capacity of NSAIDs to inhibit synthesis of prostaglandin (PG), a molecule that is synthesized by osteoblasts and autocrinally favours their growth [7, 8]. Others propose that the drug has a direct effect on the cell cycle based on *in vitro* findings that therapeutic doses of ketorolac, indomethacin, or diclofenac induce cell death in osteoblast cultures from rat foetal calvaria and may suppress bone formation and impair bone remodelling by arresting cell cycle in phase G_0_/G_1_ [9, 10].

Different NSAIDs were recently reported to affect osteoblast proliferative capacity and modulate other features of this cell population, including differentiation, antigenic profile, and phagocytic capacity [11, 12]. However, Arpornmaeklong et al. [13] found that indomethacin and celecoxib inhibit cell growth but have a less clear effect on cell differentiation as determined by alkaline phosphatase and osteocalcin synthesis.

Acetylsalicylic acid (aspirin) is very frequently administered for its anti-inflammatory, antipyretic, antiaggregant, and analgesic properties. However, in contrast to other NSAIDs, few data are available on its effects on bone tissue or on osteoblasts, the cells responsible for bone formation and regeneration. Therefore, the objective of this study was to analyse the effect of aspirin at different doses on osteoblast cell growth, using the human MG63 osteosarcoma cell line.

## 2. Materials and Methods

### 2.1. Cell Culture

 The human MG-63 osteosarcoma cell line was purchased from American Type Cultures Collection (ATCC, Manassas, VA) and maintained as described by Díaz-Rodríguez et al. [14] in Dulbecco's-modified Eagle medium (DMEM; Invitrogen Gibco Cell Culture Products, Carlsbad, CA) with 100 IU/mL penicillin (Lab Roger SA, Barcelona, Spain), 50 *μ*g/mL gentamicin (Braum Medical SA, Jaen, Spain), 2.5 *μ*g/mL amphotericin B (Sigma, St Louis, MO, USA), 1% glutamine (Sigma, St Louis, MO, USA), and 2% HEPES (Sigma), supplemented with 10% foetal bovine serum (FBS) (Gibco, Paisley, UK). Cultures were kept at 37°C in a humidified atmosphere of 95% air and 5% CO_2_. Cells were detached from the culture flask with a solution of 0.05% trypsin (Sigma) and 0.02% ethylene diamine tetra-acetic acid (EDTA) (Sigma) and then washed and suspended in complete culture medium with 10% FBS.

### 2.2. Cell Proliferation

Cell proliferation was determined by the MTT method. Osteoblasts were seeded at 1 × 10^4^ cells/mL per well into a 96-well plate without FBS and cultured at 37°C for 24 hours. Subsequently, the medium was replaced with DMEM containing aspirin at a dose of 0, 0.1, 1, 10, 20, 100, or 1000 *μ*M. At the end of treatments, the medium was replaced with DMEM containing 0.5 mg/mL MTT (Sigma) and incubated for 4 hours. Cellular reduction of the MTT tetrazolium ring resulted in the formation of a dark-purple water-insoluble deposit of formazan crystals. After incubation, the medium was aspirated and DMSO was added to dissolve the formazan crystals. Absorbance was measured at 570 nm with a spectrophotometer (Sunrise, Tecan, Männedorf, Switzerland).

### 2.3. Cell Cycle

Cultured human MG-63 osteosarcoma cells treated with 1, 10, 20, 100, or 1000 *μ*M of aspirin and untreated control cells were detached from the culture flask by treatment with a solution of 0.05% trypsin (Sigma) and 0.02% EDTA (Sigma) and subsequently washed, and suspended in PBS and prepared for the study of cell cycle as reported by García-Martínez et al. [15]. The suspension obtained was placed in 200 *μ*L PBS with 2 mL ice-cold 70% ethanol and 30% distilled H_2_O and then vigorously mixed. Cells were left for at least 30 min. in the cold and then harvested by centrifugation and resuspended in 800 *μ*L PBS. Cells were microscopically examined and, if clumped, passed through a 25-gauge syringe needle. Cells were then incubated at 37°C for 30 min. with 100 *μ*L ribonuclease (RNase) (1 mg/mL) and 100 *μ*L propidium iodide (PI). Finally, samples were analyzed by using an argon-ion laser tuned to 488 nm (Fasc Vantage Becton Dickinson), measuring forward and orthogonal light scatter and red fluorescence, determining both area and Esther peak of the fluorescent signal.

### 2.4. Apoptosis and Necrosis Analysis

Cultured human MG-63 osteosarcoma cells treated with 1, 10, 20, 100, or 1000 *μ*M aspirin for 12 h and untreated control cells were detached from the culture flask, washed and suspended in 300 *μ*L PBS and then labelled with annexin V and PI (Immunostep S.L., Salamanca, Spain). We incubated 100 *μ*L aliquots of the cell suspension with 5 *μ*L annexin V and 5 *μ*L PI for 30 min. at 4°C in the dark. Cells were then washed, suspended in 1 mL PBS, and immediately analyzed in a flow cytometer with argon laser (Fasc Vantage Becton Dickinson, Palo Alto, CA) at a wavelength of 488 nm to determine the percentage of fluorescent cells. Given that negative and positive controls were included, we calculated the percentage of annexin-positive (apoptotic) cells and PI-positive (necrotic) cells from 2000–3000 cell counts.

### 2.5. Statistical Analysis

R software (version 2.9.2, Auckland, New Zealand) was used for data analysis. Mean values (±standard deviation) were calculated for each variable. A two-way repeated-measures analysis of variance (ANOVA) was performed to examine the effects on proliferation, cell cycle, and apoptosis/necrosis induction, considering treatment (aspirin), time (24 h), and concentration (0.1, 1, 10, 20, 100, or 1000 *μ*M). When a significant interaction was identified, the Bonferroni correction was applied for planned pair-wise comparisons. *P* < 0.05 was considered significant. At least three experiments were performed for each culture.

## 3. Results

### 3.1. Effect of Aspirin Treatment on Mg-63 Cell Line Proliferation

**Figure 1:**
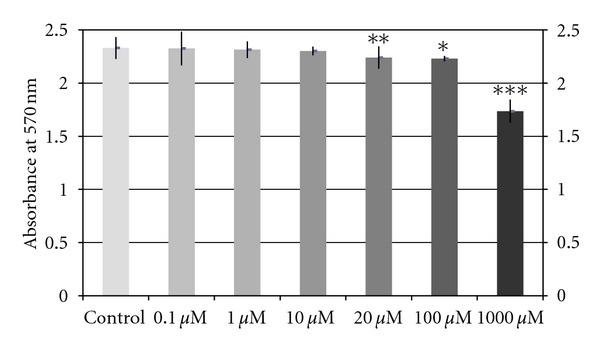
Effects of different aspirin doses (0.1 *μ*M, 1 *μ*M, 10 *μ*M, 20 *μ*M, 100 *μ*M, or 1000 *μ*M) on osteoblast proliferation in MG-63 cell line after 24 h of incubation. Data are expressed as means + SEM. We compared data between each treatment and control culture by analysis of variance (ANOVA) test. *P = 0.023, **P = 0.014, ***P = 0.001.

No significant effect on MG-63 proliferation (*P* > 0.05) was observed after 24 h treatment at any of the assayed doses (0.1, 1, and 10 *μ*M aspirin). However, significant adverse effect on cell growth was observed after treatment for 24 h at aspirin doses of 20 *μ*M (*P* = 0.014), 100 *μ*M (*P* = 0.023), and 1000 *μ*M (*P* = 0.001) (Figure 1).

### 3.2. Cell Cycle Study

**Figure 2:**
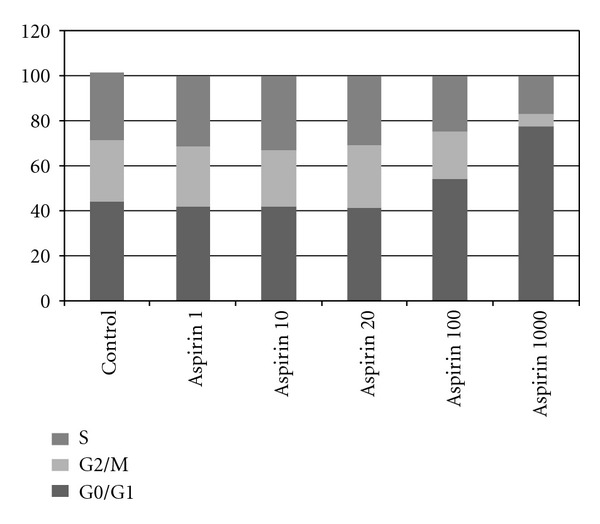
Effects of aspirin on cell cycle kinetics of MG-63 cell line determined by flow cytometry. Cultured cells were treated for 24 h with 1, 10, 20, 100, or 1000 *μ*M aspirin. Control group was not treated. G0/G1, S, and G2/M represent the percentage of cells distributed among these phases after treatment, as determined by flow cytometry. We repeated experiments at least three times.

The percentage of cells in each cell cycle phase (G0/G1, G2/M and S) was determined by flow cytometry.  Table 1 and Figure 2 depict the results of the cell cycle study. No significant effect on MG-63 cell cycle (*P* > 0.05) was observed after treatment for 24 h with 1, 10, or 20 *μ*M doses of aspirin. However, the cell percentage in G_0_/G_1_ phase was significantly increased by treatment at doses of 100 and 1000 *μ*M (*P* < 0.001). The mean percentage cell count in G_0_/G_1_ phase was 42.68 in control cultures versus 54.38 in 100 *μ*M-treated and 77.44 in 1000 *μ*M-treated cultures.

### 3.3. Apoptosis

**Figure 3:**
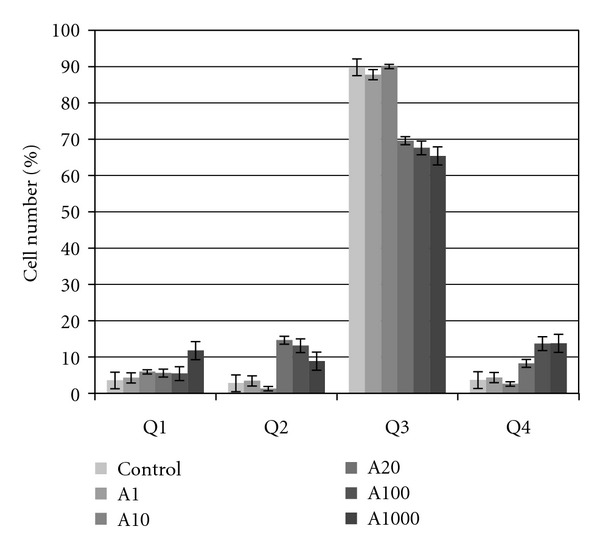
Percentage of Annexin V binding and propidium iodide uptake of MG63 cells after culture for 12 h. Q1 (necrosis: Ann V^−^, PI^+^), Q2 (late apoptosis: Ann V^+^, PI^+^), Q3 (viable cells: Ann V^−^, PI^−^), and Q4 (early apoptosis: Ann V^+^, PI^−^). Data are means ± standard deviation of at least three independent determinations.

Annexin V and PI were used to further discriminate apoptotic cell death from necrotic cell death in the cell cycle.  Table 2 and Figure 3 show results of one experiment after culture for 12 h, as an example. The number of viable cells (Ann V^−^, PI^−^) was counted in the lower left quadrant (Q3) of density plots, and the percentages of cells in early apoptosis (Ann V^+^, PI^−^, lower right quadrant Q4), late apoptosis (Ann V^+^, PI^+^, upper right quadrant Q2), and necrosis (Ann V^−^, PI^+^, upper left quadrant Q1) were determined. Figure 3 shows the cell percentage in each quadrant after 12 h exposure to aspirin at different doses. No significant effect was found on the cell percentage in any quadrant after treatment for 12 h at doses of 1 and 10 *μ*M aspirin. In contrast, a significant increase in cell percentage was observed versus controls in early apoptosis (8.31 versus 3.71%, *P* = 0.043) and late apoptosis (14.69 versus 2.84%, (*P* < 0.001) after 12 h treatment with a 20 *μ*M dose, finding a significant reduction in living cells in the treated group versus controls (69.59% versus 89.82%, *P* = 0.012). After 12 h treatment with 100 *μ*M aspirin, the cell percentage in early apoptosis increased by 13.74% (*P* = 0.002 versus controls) and the percentage in late apoptosis by 13.18% (*P* = 0.0092 versus controls) and there was a reduction of 67.61% in living cells (*P* = 0.001 versus controls). After 12 h treatment with 1000 *μ*M aspirin, cell percentage in early apoptosis increased by 13.82% (*P* = 0.001 versus controls), the percentage in late apoptosis increased by 13.18% (*P* = 0.0092), and the percentage of necrotic cells increased by 11.81% (*P* = 0.001), and there was a 65.41% decrease in living cells (*P* = 0.002).

## 4. Discussion

The anti-inflammatory properties of NSAIDs, including aspirin, derive from their powerful inhibitory effects on cyclooxygenase metabolites such as prostaglandin E2 [16]. Consequently, some authors attribute the adverse effect of NSAIDs on osteoblasts to a reduced synthesis of prostaglandins, which act as bone cell growth factors, as a result of the inhibition of cyclooxygenase enzymes [17]. However, there is some controversy concerning the adverse effect of NSAIDs on osteoblast growth [10, 18]. The disparate results obtained may be due to multiple factors, including dosage, treatment duration, and the species in question [13, 17].

In this study, the proliferative capacity of cells from the MG-63 cell line was not significantly inhibited by 24 h treatment with 0.1, 1, or 10 *μ*M aspirin but was significantly inhibited by 24 h treatment with 20, 100, and 1000 *μ*M aspirin. Therefore, this NSAID does not have an adverse effect on bone cell proliferation *in vitro *at the therapeutic dose range used in the clinical setting (1 to 10 *μ*M). It was previously reported that the dose of NSAIDs is a key factor in the effect on cell proliferation as well as on cell differentiation and migration [19].

The effect of different doses on the growth of the MG63 line is closely related to the effects observed on the cell cycle. Thus, the negative effect on growth at 100 and 1000 *μ*M aspirin may be explained by the significant increase in the percentage of cells in phase G0/G1 versus the control group. Ann V and PI staining showed that the reduced cell growth at 20 and 100 *μ*M doses was attributable to apoptosis rather than necrosis. In contrast, treatment with a 1000 *μ*M produced an increase in necrotic cells as well as cells in apoptosis.

We highlight that treatment of the MG63 line with therapeutic doses of aspirin (1–10 *μ*M) did not cause cell death, cell cycle changes, or modifications in apoptosis/necrosis induction. In contrast, therapeutic doses of ketorolac, indomethacin, or diclofenac induced cell death in osteoblast cultures from rat foetal calvaria and may suppress bone formation and impair bone remodelling by cell cycle arrest in G_0_/G_1_ phase [9, 10]. In another *in vitro* study, 24 h treatment with indomethacin or nimesulide at therapeutic doses also reduced osteoblast growth capacity and produced significant changes in the cell cycle, which was again arrested in G_0_/G_1_ phase [12].

A recent cohort study of subjects exposed and not exposed to NSAIDs and opioids observed no difference in bone mineral density over time but reported excessive risk of fractures in users of some NSAIDs, although not in those receiving aspirin [20]. These results are consistent with the present *in vitro* data.

Furthermore, another important aspect of aspirin, from a clinical standpoint, is that when the lipoxin pathway is activated in the presence of aspirin, acetylation of the cyclooxygenase 2 enzyme present at inflammation sites not only inhibits further production of prostanoids, but also induces the synthesis of 15-hydroxyeicosatetraenoic acid, which is then transformed into 5(S)-epoxytetraene in leukocytes in the presence of 5-lipoxygenase. The 5(S)-epoxytetraene intermediate is then transformed to 15-epilipoxins or aspirin-triggered lipoxins, which are more bioactive than native lipoxins and have more powerful resolving properties [21, 22].

In conclusion, aspirin at therapeutic doses appears to have no adverse effect on osteoblast growth, unlike some other NSAIDs. Growth was only reduced at higher doses, by cell cycle arrest and apoptosis induction. These findings support the use of aspirin in the treatment of inflammation and pain in bone lesions requiring tissue regeneration. Given reports that indomethacin and diclofenac can increase cell adhesion and reduce the migration of different cell populations [23–25], further research is warranted to determine whether aspirin may exert action on the cell adhesion or migration of osteoblasts, other key factors in the regenerative process.

## Figures and Tables

**Table 1:** Numerical data for cell cycle fluorescence profile of MG-63 cells in culture treated for 24 h with aspirin doses of 1, 10, 20, 100, or 1000 *μ*M.

	G0-G1		G2-M		S	
	Mean	*P* value	Mean	*P* value	Mean	*P* value
Control	44.34 (2.79)	—	27.22 (0.57)	—	30.09 (0.07)	—
Aspirin 1 *μ*M	42.34 (2.63)	0.46	26.41 (2.26)	0.58	31.02 (0.41)	0.07
Aspirin 10 *μ*M	42.21 (5.25)	0.5	24.85 (3.74)	0.3	32.94 (2.48)	0.1
Aspirin 20 *μ*M	41.54 (2.16)	0.24	27.52 (0.60)	0.57	30.93 (1.75)	0.47
Aspirin 100 *μ*M	54.38 (1.79)	0.006*	20.94 (1.35)	0.002*	24.68 (2.96)	0.03
Aspirin 1000 *μ*M	77.44 (0.83)	0.0001*	5.95 (1.15)	0.0001*	16.60 (1.93)	0.0001*

*Significant differences.

**Table 2:** Numerical data for the percentage annexin V binding and propidium iodide uptake of MG63 cells after culture for 12 h. Q1 (necrosis: Ann V^−^, PI^+^), Q2 (late apoptosis: Ann V^+^, PI^+^), Q3 (viable cells: Ann V^−^, PI^−^), Q4 (early apoptosis: Ann V^+^ PI^−^). Data are means ± standard derivation of at least three independent determinations.

		MEAN	SD	*P*	C.I. (95%)
Control	Necrosis	3.61	1.6163	—	—
	Late Ap.	2.84	1.4530	—	—
	Negative	89.82	4.2866	—	—
	Apoptosis	3.71	1.9480	—	—

A1 *μ*M	Necrosis	9.31	0.5695	0.005	−8.4471; −2.9528
	Late Ap.	8.16	0.4923	0.004	−7.7793; −2.8606
	Negative	73.09	0.3157	0.021	6.1649; 27.3016
	Apoptosis	9.42	0.3005	0.007	−8.8695; −2.5504

A10 *μ*M	Necrosis	5.97	0.5697	0.076	−5.1072; 0.3872
	Late Ap.	1.3467	0.2514	0.213	−1.9720; 4.9720
	Negative	90.0267	1.3050	0.941	−7.3861; 6.9794
	Apoptosis	2.6533	0.6192	0.421	−2.2166; 4.3332

A20 *μ*M	Necrosis	5.64	1.4516	0.182	−5.5058; 1.4592
	Late Ap.	14.69	0.4531	0.000	−14.2832; −9.4034
	Negative	69.59	0.8826	0.012	10.1319; 30.3281
	Apoptosis	8.31	1.8938	0.043	−8.9583; −0.2482

A100 *μ*M	Necrosis	5.48	1.4807	0.215	−5.3772; 1.6505
	Late Ap.	13.18	2.1219	0.002	−14.4558; −6.2108
	Negative	67.61	2.3404	0.001	14.3844; 30.0422
	Apoptosis	13.74	3.0390	0.009	−15.8230; −4.2502

A1000 *μ*M	Necrosis	11.81	1.5383	0.003	−11.7735; −4.6197
	Late Ap.	8.94	3.2706	0.042	−11.8335; −0.3597
	Negative	65.41	3.5840	0.002	15.4499; 33.3633
	Apoptosis	13.82	1.0186	0.001	−13.6404; −6.5929
